# The Seventh Ohio District Dental Society

**Published:** 1889-10

**Authors:** 


### The Seventh Ohio District Dental Society.

This society, which was organized in Dayton in May last, held its first regular meeting at the Lincoln Club Rooms, on Tuesday, September 17th, at 10 o'clock A. M., and continued during the day. The attendance was good for the first meeting, and an interest was manifested that portends well for the future; several papers were read, and interesting discussions were had upon them, and upon the whole it was a profitable meeting for those who were present.

Action was taken for securing a complete Roster of the dentists of the district. This is an important matter and will doubtless be followed up by every district society in the State.

The "Seventh" will be able to make a good report to the next meeting of the State Society. The hope was entertained that each of the eight district societies might be organized before the annual meeting of the State Society; it is doubtful whether this will be done or not; but it is desirable that it be not deferred longer than is necessary.

